# Patterns of failure after multimodal treatments for high-grade glioma: effectiveness of MIB-1 labeling index

**DOI:** 10.1186/1748-717X-7-104

**Published:** 2012-06-26

**Authors:** Kazuyuki Uehara, Takashi Sasayama, Daisuke Miyawaki, Hideki Nishimura, Kenji Yoshida, Yoshiaki Okamoto, Naritoshi Mukumoto, Hiroaki Akasaka, Masamitsu Nishihara, Osamu Fujii, Toshinori Soejima, Kazuro Sugimura, Eiji Kohmura, Ryohei Sasaki

**Affiliations:** 1 Division of Radiation Oncology, Kobe University Graduate School of Medicine, Kobe, Hyogo, Japan; 2 Department of Neurosurgery, Kobe University Graduate School of Medicine, Kobe, Hyogo, Japan; 3 Department of Radiation Oncology, Osaka Police Hospital, Tennoji, Japan; 4 Division of Radiation Oncology, Kobe University Graduate School of Medicine, Akashi, Hyogo, Japan

**Keywords:** Pattern of failure, Glioblastoma, Radiotherapy, MIB-1 labeling index

## Abstract

**Background:**

The purpose of the present study was to analyze the recurrence pattern of high-grade glioma treated with a multimodal treatment approach and to evaluate whether the MIB-1 labeling index (LI) could be a useful marker for predicting the pattern of failure in glioblastoma (GB).

**Methods and materials:**

We evaluated histologically confirmed 131 patients with either anaplastic astrocytoma (AA) or GB. A median dose was 60 Gy. Concomitant and adjuvant chemotherapy were administered to 111 patients. MIB-1 LI was assessed by immunohistochemistry. Recurrence patterns were categorized according to the areas of recurrence as follows: central failure (recurrence in the 95% of 60 Gy); in-field (recurrence in the high-dose volume of 50 Gy; marginal (recurrence outside the high-dose volume) and distant (recurrence outside the RT field).

**Results:**

The median follow-up durations were 13 months for all patients and 19 months for those remaining alive. Among AA patients, the 2-year progression-free and overall survival rates were 23.1% and 39.2%, respectively, while in GB patients, the rates were 13.3% and 27.6%, respectively. The median survival time was 20 months for AA patients and 15 months for GB patients. Among AA patients, recurrences were central in 68.7% of patients; in-field, 18.8%; and distant, 12.5%, while among GB patients, 69.0% of recurrences were central, 15.5% were in-field, 12.1% were marginal, and 3.4% were distant. The MIB-1 LI medians were 18.2% in AA and 29.8% in GB. Interestingly, in patients with GB, the MIB-1 LI had a strong effect on the pattern of failure (P = 0.014), while the extent of surgical removal (P = 0.47) and regimens of chemotherapy (P = 0.57) did not.

**Conclusions:**

MIB-1 LI predominantly affected the pattern of failure in GB patients treated with a multimodal approach, and it might be a useful tool for the management of the disease.

## Background

Glioblastoma (GB) is one of the most aggressive primary brain tumors. The standard treatment includes a multimodal approach with surgery, radiotherapy, and chemotherapy. Although concurrent chemoradiotherapy with temozolomide has been shown to have a survival benefit for GB [1], the overall outcome for GB has not improved significantly, and intracranial tumor recurrence or progression develops in most patients in less than 1 year.

"The nature of GB, with widespread tumor infiltration and lower radiosensitivity, has frustrated efforts to provide durable tumor control with radiotherapy." Although several investigators demonstrated the presence of widespread microscopic infiltration within the brain [2], local recurrence predominates with this disease, most often within 2 cm of the original tumor. Conformal radiotherapy that includes postoperative peritumoral edema in the target volume is currently being used in the Radiation Therapy Oncology Group (RTOG) trials (e.g., the RTOG 0525 and RTOG 8525 trials). Alternatively, treatment strategies based on contrast-enhancement of the tumor (using CT/MRI) plus 2-cm margins is currently employed in many European institutions, according to the European Organization for Research and Treatment of Cancer (EORTC) recommendations [3-6]. Therefore, consensus guidelines for clinical target volume (CTV) delineation remain controversial. On the contrary, with respect to radiation doses, although prescribed doses of 60 Gy are generally employed, attempts at dose escalation or altered fractionation of radiotherapy have been widely challenged [7,8]. Dose escalation to 90 Gy [7] or the addition of a stereotactic radiosurgery (SRS) boost [9] may reduce the failure rates in the high-dose region, although recurrence then tends to occur just outside the high-dose region.

Determination of proliferative activity using the monoclonal antibody MIB-1 labeling index (LI) has been investigated in malignant gliomas [10,11]. Although some studies indicated that MIB-1 LI was correlated with an increasing grade of malignancy, no study has evaluated whether MIB-1 LI might predict a pattern of failure in GB patients.

We evaluated the outcome of high-grade glioma treated with a multimodal approach, and investigated patterns of failure with regard to MIB-1 LI. The purpose of this study was to analyze the recurrence patterns and to evaluate whether MIB-1 LI could be useful as a marker to predict the pattern of failure in high-grade glioma.

## Materials and methods

### Patients

In this study, 142 consecutive patients with histologically proven high-grade glioma, i.e., anaplastic astrocytoma (AA) or GB, were retrospectively reviewed. These patients were treated with radiotherapy at Kobe University Hospital or Hyogo Cancer Center between 2000 and 2010. The retrospective review and the use of clinical data were approved by the institutional ethics board. Eleven patients were not included in the analysis: 5 patients who were treated with palliative therapy, 5 patients who discontinued the course of radiotherapy, and 1 patient who refused to enroll in the study. Therefore, a total of 131 patients were included in the analysis. Written informed consent was obtained from the patient for publication of this case report and any accompanying images.

### Extent of surgical removal

The extent of surgical removal was assessed using surgical information and/or the comparison of preoperative and postoperative magnetic resonance imaging (MRI). The extent of surgical removal was categorized into 4 subgroups: gross total removal (GTR), subtotal removal (STR), partial removal (PR), and biopsy. GTR was defined as a resection of more than 99% of tumor volume; STR, a resection of 80–99% of tumor volume; PR, as a resection of 20–80% of tumor volume; and biopsy, a resection of less than 20% of tumor volume.

### Methods of radiation therapy

Radiotherapy was started 2–4 weeks after surgery. Treatment planning computerized tomography (CT) scans obtained images at 2.5–5.0-mm slice intervals. Information obtained in the simulation CT scan was combined with any available MRI data, including post-contrast T1- and T2-weighted images or fluid-attenuated inversion recovery (FLAIR) images as fusion images. For the planning of radiotherapy, FOCUS (2000–2004) or Xio software (2005–2010) (CMS Co. Ltd., Japan) was used. The gross tumor volume (GTV) was carefully defined considering gadolinium-enhanced lesions in presurgical MRI and/or residual tumor lesions in post-surgical MRI. In our methods, the GTV was basically defined based on pre-surgical tumor extents. However, in case that range and portion of surgical cavities was beyond the pre-surgical tumor extents. The GTV were reconstructed by pre-surgical tumor extents combined with post-surgical imaging, because to set GTV from only presurgical images was considered to be insufficient. The CTV for the initial plan was delineated, including the area of peritumoral edema (high-intensity lesion on FLAIR images) plus the 1.5–2.0-cm margin, and the CTV-boost was defined based on the GTV with a 1.5–2.0-cm margin. For the setup margin, a 5-mm margin was applied to each CTV (CTV-initial, CTV-boost) to create PTVs (PTV-initial, PTV-boost). All patients were treated with conventional fractions of 1.8–2.0 Gy 5 times a week. A median total dose of 60 Gy (range: 54–71.2 Gy) was delivered in 27–39 fractions with concomitant and adjuvant chemotherapy. The prescribed dose was 40–50.4 Gy to the PTV-initial for both AA and GB, followed by 10–20.8 Gy to the PTV-boost. The 100% isodose line was defined at the isocenter, and the dose was prescribed to this point. All patients were treated with three-dimensional conformal radiation therapy consisting of 3–5 non-coplanar fields. The normal tissues delineated included the optic nerves, optic chiasm, brainstem, eye, and optic lens. The optic nerve and optic chiasm maximum doses were restricted to ≤50 Gy. The maximum dose to the brainstem was restricted to ≤54 Gy.

### Chemotherapy

Regimens of chemotherapy shifted according to the study period. Between September 2000 and September 2006, 55 patients received a combination regimen that consisted of ACNU (nimustine), vincristine, and interferon with radiotherapy. After October 2006, 56 patients were scheduled to receive temozolomide concurrent with radiotherapy at a dosage of 75 mg/m^2^/day followed by adjuvant therapy at a dosage of 150–200 mg/m^2^/day for 5 days every 28 days, unless the disease progressed or the patient experienced treatment-related toxicity or declining performance status necessitating the discontinuation of chemotherapy. In our policy, concomitant chemotherapy was considered to all patients with AA and with GB. However, 4 (15%) patients with AA could not receive chemotherapy because of their poor general conditions. Thus, 111 (84.7%) patients received chemotherapy.

### Immunohistochemical analysis

Paraffin-embedded tissue blocks were sectioned (4-μm thick) onto slides and then deparaffinized. Sections were immunostained using the Vectastain ABC Elite Kit (Vector Laboratories, Burlingame, CA, USA) according to the manufacturer’s instructions with anti-MIB-1 monoclonal antibody. The MIB-1 LI was calculated as the percentage of positively stained tumor cell nuclei among 1000 nuclei detected in areas with the greatest degree of immunostaining [11]. A median of the MIB-1 LI was used as reference to establish a cut off point [12].

### Recurrence diagnosis methods

The diagnosis of tumor recurrence and disease progression was made using MRI. Positron emission tomography (PET) imaging was not routinely used. Recurrence was defined as follows: central failure if more than 95% of the recurrence volume was in the 95% isodose line of 60 Gy (boost volume); in-field if more than 95% of the recurrence volume was in the high-dose volume (50 Gy); marginal when less than 95% of recurrence volume was outside the high-dose volume; distant when recurrence was outside the RT field (<20% isodose line).

### Assessment of the pattern of failure

There were 43 patients (8 AA patients and 35 GB patients) who showed no sign of recurrence. Among 88 patients who experienced recurrence, 14 patients’ samples were not submitted for immunohistochemical evaluation because of insufficient samples. In total, data from 74 patients (16 AA patients and 58 GB patients) were evaluated for the pattern of failure in this study.

### Follow-up evaluation and statistical analyses

In the follow-up evaluations, local and systemic tumor control was assessed at 1–3-month intervals using MRI. Progression-free survival (PFS) and overall survival (OS) rates were analyzed statistically in all patients. PFS was calculated from the first day of radiotherapy to the date of any recurrence or death, or censored the date of the last follow-up. OS was calculated from the first day of radiotherapy to the date of death, or censored the date of the last follow-up, and calculations were made using Kaplan–Meier estimates. Statistical significance was determined using the log-rank test. The chi-square test was used for the comparisons among cohorts in this analysis. Variables influencing OS and PFS were evaluated with multivariate Cox proportional hazards model with a 95% confidence interval. Differences were considered statistically significant at P values < 0.05. All statistical analyses were performed using StateView (version 5.0).

## Results

### Patients and treatments

Patient and treatment details are shown in Table 1. Twenty-six (20%) patients had AA, and 105 (80%) patients had GB. There were no statistically significant differences between the 2 cohorts with regard to radiotherapy methods, the extent of surgical removal, or chemotherapy regimens.

**Table 1:** Patient characteristics and treatments (n = 131)

**Characteristics**		**Anaplastic astrocytoma**	**Glioblastoma**
		**n = 26 (%)**	**n = 105 (%)**
Age			
	Median	52.5 years	59 years
	(range)	(18–75 years)	(16–77 years)
Sex			
	Male	14 (54)	59 (56)
	Female	12 (46)	46 (44)
Original tumor location			
	Frontal lobe	11 (42)	36 (34)
	Temporal lobe	5 (19)	35 (33)
	Parietal lobe	3 (12)	20 (19)
	Thalamus	4 (15)	3 (3)
	Occipital	1 (4)	6 (6)
	Cerebellum	2 (8)	2 (2)
	Basal ganglia	0 (0)	2 (2)
	Brainstem	0 (0)	1 (1)
MIB-1 labeling index			
	Median (range)	18.2% (2–35)	29.8% (2–80)
Radiotherapy			
	Median dose	60 Gy	60 Gy
	(range)	(54–68.4 Gy)	(54–71.2 Gy)
Extent of surgical removal			
	Gross total removal	3 (12)	18 (17)
	Subtotal removal	5 (19)	15 (14)
	Partial removal	11 (42)	59 (56)
	Biopsy only	7 (27)	13 (12)
Chemotherapy			
	ACNU (Nimustine)	11 (42)	44 (42)
	Temozolomide	11 (42)	45 (43)
	None	4 (15)	14 (13)
	Unknown	0 (0)	2 (2)

### Progression-free and overall survival

**Figure 1:**
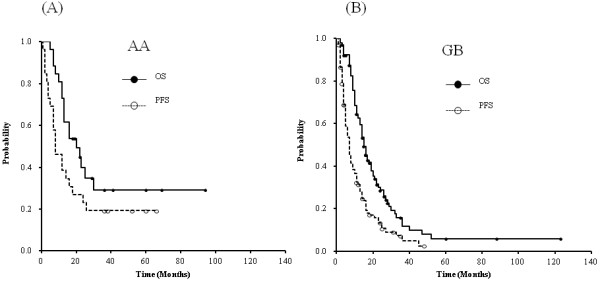
**Kaplan-Meier curves of overall survival and progression-free survival for each histological grade.** Kaplan-Meier curves of progression-free survival (PFS) and overall survival (OS) according to histology grade: (**A**) anaplastic astrocytoma (n = 26), (**B**) glioblastoma (n = 105).

The median follow-up periods were 13 months for all patients and 19 months for those remaining alive. Among AA patients, the 2-year PFS and OS rates were 23.1% and 39.2%, respectively, while in GB patients, the rates were 13.3% and 27.6%, respectively (Figure 1). The median survival times (MSTs) were 20 months in patients with AA and 15 months in patients with GB. The cause of death among AA patients was identified as primary tumor deterioration in 17 patients (100%), while causes of death in GB cases were identified as primary tumor deterioration in 75 patients (93.8%), other diseases (pneumonia) in 4 patients (5%), and unknown in one patient (1.2%). Among 105 patients with GB, except for 16 patients who did not receive chemotherapy or unknown, 44 (42%) patients were treated with ACNU-based regimens during 2000 to September 2006, and 45 (43%) patients were treated with TMZ-based regimens in between October 2007 to thereafter (Table 1). Between ACNU-based and TMZ-based groups with GB, there were no significant difference in OS (P =0.86) and PFS (P =0.42), and in. In patients with AA, the tendency was similar, resulting in OS (P =0.93) and PFS (P =0.72).

### Patterns of failure for AA or GB

**Figure 2:**
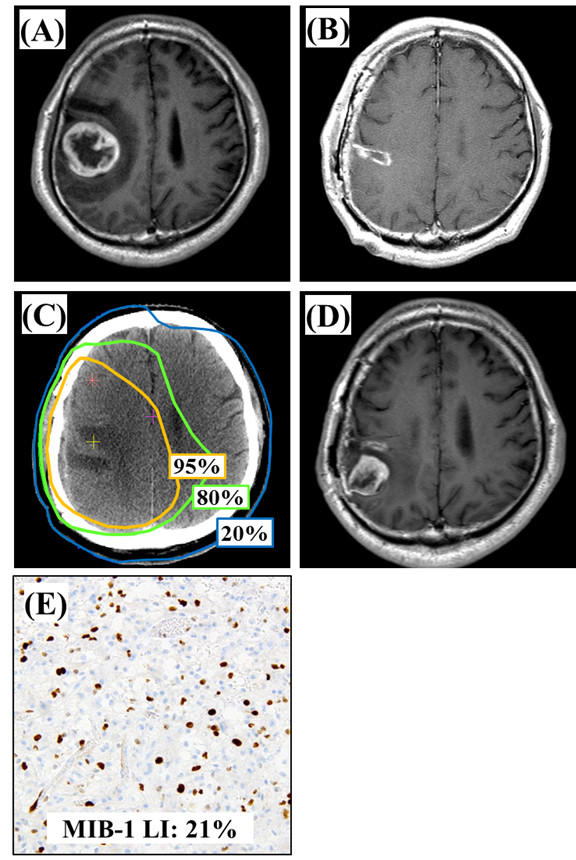
**Representative images for the case of GB with lower MIB-1 LI.** A 76-years female patient with GB showing a lower MIB-1 LI (21%) that recurred in the central region. (**A**) A postcontrast MR image (T1 weighted) before surgery. (**B**) A postcontrast MR image (T1 weighted) after a gross total removal. (**C**) Treatment planning CT image showing the 95% isodose curve (yellow), the 80% isodose curve (green), and the 20% isodose curve (blue). (**D**) A postcontrast MR image (T1 weighted) at 4 months after completing radiotherapy showing a recurrence tumor that developed in the central. (**E**) Immunohistochemical analyses (×200).

**Figure 3:**
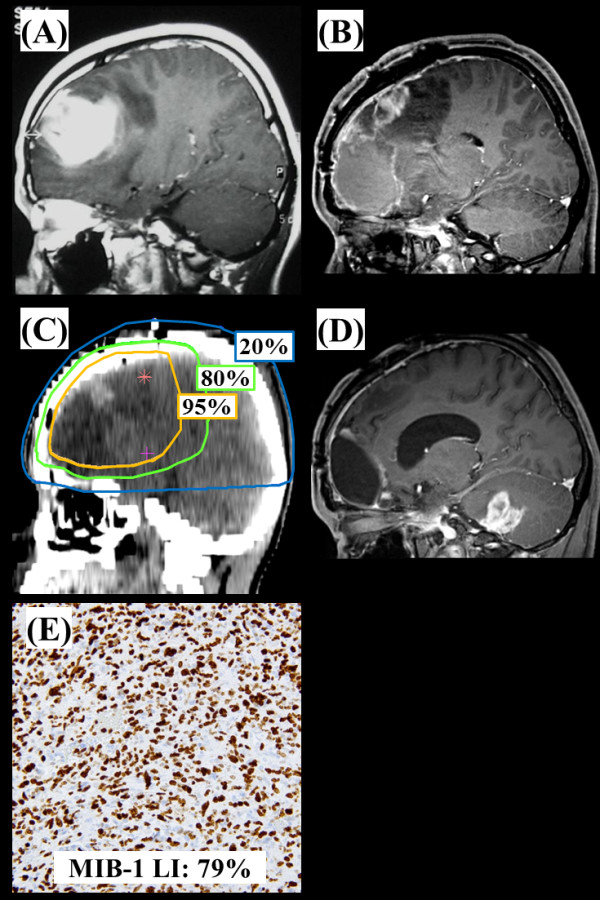
**Representative images for the case of GB with higher MIB-1 LI.** A 24-years male patient with GB showing a higher MIB-1 LI (79%) that recurred in the distant region. (**A**) A postcontrast MR image (T1 weighted) before surgery (**B**) A postcontrast MR image (T1 weighted) after a gross total removal. (**C**) Treatment planning CT image showing the 95% isodose curve (yellow), 80% isodose curve (green), and the 20% isodose curve (blue). (**D**) A postcontrast MR image (T1 weighted) at 11 months after completion of radiation showing a recurrence tumor that developed in the distant. (**E**) Immunohistochemical analyses (×200).

Among the AA patients, 11 (68.7%) displayed recurrence in the central area, while 3 (18.8%) displayed recurrence in the in-field area and 2 (12.5%), in the distant area (Table 2). Among 5 patients who displayed recurrence outside of the central area, 1 (20%) patient also had a recurrent tumor in the central area. In contrast, among patients with GB, 40 (69.0%) displayed recurrence in the central area, while 9 (15.5%) displayed recurrence in the in-field area; 7 (12.1%), in the marginal area; and 2 (3.4%), in the distant area (Table 2). Among those 18 patients whose recurrence was outside of the central area, 6 (33%) patients also had a recurrent tumor in the central area. Representative cases of recurrent tumors observed in the central and distant areas are shown in Figure 2 and Figure 3, respectively.

**Table 2:** Patterns of recurrence after multimodal treatments (n = 74)

**Recurrent sites**	**Anaplastic Astrocytoma**	**Glioblastoma**
	**n = 16 (%)**	**n = 58 (%)**
Central	11 (68.7)	40 (69.0)
In-field	3 (18.8)	9 (15.5)
Marginal	0 (0)	7 (12.1)
Distant	2 (12.5)	2 (3.4)

### Distribution of MIB-1 LI

**Figure 4:**
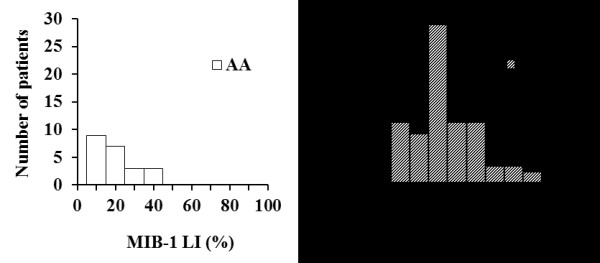
**Distributions of MIB-1 LI.** Distributions of MIB-1 LI in patients with AA (n = 22) and in patients with GB (n = 79).

MIB-1 LI scores were available in 101 patients (22 AA patients and 79 GB patients). The MIB-1 LI of AA was comparatively low, and the median MIB-1 LI was 18.2% (range: 2–35%). In contrast, the MIB-1 LI of GB was higher and more widely distributed. The median MIB-1 LI was 29.8% in GB (range: 2–80%) (Figure 4).

### MIB-1 LI correlation with patterns of failure in GB

Of the 131 patients, 101 patients (16 AA patients and 58 GB patients) were available for evaluation of recurrence pattern and MIB-1 LI. Other 27 patients had not yet shown signs of tumor recurrence. Among the GB patients, recurrence occurred central area and others in 25 (83%) and 5 (17%) of patients with the MIB-1 LI <30%, and in 15 (54%) and 13 (46%) of patients with the MIB-1 LI ≥ 30%, respectively (P = 0.014) (Table 3) In contrast, among the AA patients, recurrence occurred central area and others (in-field, marginal, or distant) in 9 (69%) and 4 (31%) of patients with the MIB-1 LI < 30%, and in 2 (67%) and 1 (33%) of patients with the MIB-1 LI ≥ 30%, respectively (P = 0.931).

**Table 3:** Factors may or may not affect the patterns of failure in patients with glioblastoma or anaplastic astrocytoma

**Patients with glioblastoma**									
	**MIB-1 (n =58)**			**Extent of surgical removal (n =58)**			**Chemotherapy (n =53)**		
Sites of	< 30	≧ 30	*P* value	> 80%	< 80%	*P* value	ACNU	TMZ	*P* value
recurrence							based	based	
	n = 30	n = 28		n = 20	n = 38		n = 23	n = 30	
Central	25	15	0.014	15	25	0.47	17	20	0.57
Others *	5	13		5	13		6	10	
Patients with anaplastic astrocytoma									
	**MIB-1 (n =16)**			**Extent of surgical removal (n =16)**			**Chemotherapy (n =13)**		
Sites of	< 30	≧ 30	*P* value	> 80%	< 80%	*P* value	ACNU	TMZ	*P* value
recurrence									
							based	based	
	n = 13	n = 3		n = 5	n = 11		n = 5	n = 8	
Central	9	2	0.93	3	8	0.61	4	6	0.84
Others *	4	1		2	3		1	2	

* Others = In-field + marginal + distant.

^†^ > 80% = GTR or Sub-total, < 80% = Partial/Biopsy.

For patients with GB whose MIB-1 LI was < 30%, 25 (83%) developed a central recurrence, whereas 15 (54%) for those with MIB-1 LI ≥ 30% (P = 0.014). Among patients with the MIB-1 LI < 30% who recurred in the other sites, there were 2 (7%) patients in the in-field, 3 (10%) patients in the marginal, and none in the distant, whereas those with MIB-1 LI ≥ 30%, there were 7 (25%) patients in the in-field, 4 (14%) patients in the marginal, and 2 (7%) patients in the distant, respectively.

In patients with GB, the MIB-1 LI was strongly affected by the pattern of failure (P = 0.014), while the extent of surgical removal (P = 0.47) or regimens of chemotherapy (P = 0.57) were not (Table 3). In contrast, in patients with AA, the MIB-1 (P = 0.93), extent of surgical removal (P = 0.84), and regimens of chemotherapy (P = 0.61) did not influence the pattern of failure.

### Evaluation of prognostic factor for OS and PFS

Results of univariate and multivariate analysis of prognostic factors were shown (Table 4). In the multivariate analysis, age (P = 0.04), performance status (P < 0.01) and extent of resection (P = 0.04) were significant prognostic factors for OS. In case of PFS, performance status (P = 0.01) was the single significant prognostic factor. Om the contrary, MIB-1 LI was not significant factor for OS or PFS in both univariate and multivariate analyses.

**Table 4:** Results of univariate and multivariate analyses for OS and PFS in patients with glioblastoma or anaplastic astrocytoma

	**Variable**	**Test for favorable status**	**OS**	**PFS**
Univariate analysis			P value	P value
	PS	0,1 (n = 60) vs. 2,3, and 4 (n = 60)	<0.01	<0.01
	Age	< 50 (n = 37) vs. ≧ 50 (n = 94)	0.04	0.08
	Extent of resection	GTR/STR (n = 41) vs. PR/biopsy (n = 90)	0.06	0.37
	Histological grade	AA (n = 26) vs. GB (n = 105)	0.08	0.11
	MIB-1 LI	< 30 (n = 61) vs. ≧ 30 (n = 43)	0.21	0.59
	BED or Radiation dose	≤ 72 GyE (n = 77) vs. > 72 GyE (n = 54)	0.29	0.53
	Gender	Male (n = 73) vs. Female (n = 58)	0.73	0.54
	Chemotherapy (CT)	ACNU based (n = 55) vs. TMZ based (n = 56)	0.91	0.45
Multivariate analysis				
	PS		<0.01	0.01
	Age		0.04	0.07
	Extent of resection		0.04	0.30
	MIB-1 LI		0.07	0.12

## Discussion

In the management of GB, the pattern of failure is one of the major concerns in relation to the CTV margins, optimal radiation dose, and identification of biomarkers. However, the factors affecting the pattern of failure in GB have not been described in detail. The present study is the first to report an association between the MIB-1 LI and the pattern of failure in patients with GB.

In prior studies, treatment factors, including CTV margins, radiation dose, and chemotherapy, were widely discussed as the most important aspects determining the pattern of failure of GB [3-5,7,13-15]. Chang et al. [3], in a study based on protocols following RTOG guidelines, conducted at the M.D. Anderson Cancer Center, reported similar patterns of failure in a series of 48 GB patients by comparing treatment plans based on residual tumor and resection cavity plus 2-cm margins. Liang et al. [4] studied 42 patients with malignant glioma irradiated with 60 Gy using conformal radiation techniques. All patients developed recurrences within 2 cm of the original tumor. Lee et al. [5] analyzed 36 patients with high-grade astrocytoma treated with 70 Gy and 80 Gy to a CT/MRI-localized PTV (tumor size: <2–3 cm) and found that 89% of the recurrences occurred with a central or in-field recurrence pattern. McDonald et al. [13] demonstrated that through the use of limited margins, 92% of the patients in their series had a PTV boost margin of 1 cm or less, only 5% of patients had a marginal failure, and 2% patients had a distant failure with temozolomide chemoradiation. Minniti et al. [14] demonstrated that the majority of patients treated with RT plus concomitant and adjuvant temozolomide have central recurrences, while distant new lesions may occur in more than 10% of patients. Milano et al. [15] demonstrated that central recurrence of glioblastoma treated with radiation and temozolomide predominates and persists over time, whereas new in-field, marginal, and distant recurrences commonly develop, particularly at later time points in patients with longer survival. Our results regarding the proportions of particular sites of recurrence were consistent with those reports (Table 5). Although the reports cited above included different CTV margins, radiation doses, and regimens of chemotherapy, the patterns of failure of GB appear to be similar, suggesting that these treatment factors might not strongly affect the pattern of failure in GB.

**Table 5:** Comparison of published data with regard to patterns of failure in patients with glioblastoma

**Author**	**Year**	**Number**	**Treatment**	**Dose (Gy)**	**Margin (cm)**	**Recurrence sites**			
						**Central**	**In-field**	**Marginal**	**Distant**
Nakagawa [7]	1998	38	3DCRT + ACNU	60–80	0–2	90%^†^			5%
				90	0–2	46%^††^			8%
Lee [5]	1999	36	3DCRT	70–80	1.5	72%	17%	8%	3%
Chan [6]	2002	34	3DCRT	90	0.5	78%	13%	9%	
Chang [3]	2007	48	3DCRT ± chemo^*^	60	1	83%	6%	6%	4%
Brandes [16]	2009	95	3DCRT + TMZ	60	2–3	72%		6%	22%
Milano [15]	2010	54	3DCRT + TMZ	60	2–2.5	92%^§^		15%	13%
Minniti [14]	2010	105	3DCRT + TMZ	60	1–2	79%	6%	6%	14%
McDonald [13]	2011	41	(IMRT or 3DCRT) ± TMZ	60	0.8	78%	15%	5%	2%
This study	2012	58	3DCRT ± (ACNU or TMZ)	60	1.5–2	69%	16%	12%	3%

*21/48 patients received adjuvant or concurrent chemotherapy (carmustine, procarbazine, and temozolomide).

†5% were subependymal recurrences (did not apply to our classification method of recurrence sites).

††46% were subependymal recurrences (did not apply to our classification method of recurrence sites).

§Insufficient data to apply to our classification method of recurrence sites.

Several clinical values of MIB-1 LI have been reported in human gliomas [10,11,17,18]. Johnnessen and Torp [10] evaluated the clinical usefulness of MIB-1 LI in grading for glioma, based on a review of 16 studies including a total of 915 patients. In addition, MIB-1 LI can be considered as an important proliferation parameter, and may be associated with clinical growth parameters independent of other prognostic factors in gliomas. Several cut-off studies have observed a significant correlation between MIB-1 LI and postoperative survival in astrocytic gliomas [17]. However, the majority of studies to date have failed to confirm such an association between the MIB-1 LI and survival in patients with GB [18]. In our series, values of the MIB-1 LI were widely distributed in both GB and AA cohorts. Median of the distribution in GB (cut off = 30) were first evaluated for correlation with patterns of failure, and other cut off points (cut off = 10, 20, 35, and 50) were also assessed. Among those cut off points, the cut off = 30 was found to be the only significant value as for the pattern of failure in GB. On the contrary, any cut-off point did not have useful values in AA. The discrepancy between GB and AA might be a matter of debate. It is at least speculated that, in our series, the distribution of the MIB-1 LI in AA was much smaller (2 - 35%) than that in GB (2 - 80%). Therefore, to set cut off might be difficult. Of course, other possibilities might be raised. This issue should be discussed more with accumulation of reports.

Notably, our results indicated that the MIB-1 LI affected the pattern of failure in patients with GB, while the extent of surgical removal or chemotherapy regimens did not (Table 3). Previously, Shibamoto et al. [19] demonstrated that extent of surgery was associated with the prognosis of GB in their analysis of 178 cases. However, there are no reports demonstrating that MIB-1 LI can provide clues for the recognition of the pattern of failure in patients with high-grade glioma. Thus, to our knowledge, this report is the first to show the clinical value of the MIB-1 LI in GB. On the contrary, in patients with AA, no association between MIB-1 and the pattern of failure was observed. Several reasons may explain this result. Because AA is less aggressive than GB, MIB-1 LI did not reflect disease behavior in AA. In addition, the number of patients with AA was quite small. Therefore, although our findings might be restricted to GB patients, they are valuable in elucidating novel aspects of GB. Because MIB-1 LI is known to be influenced by both the staining and counting methods, inter-laboratory and inter-observer variability need to be carefully determined [20]. Moreover, in addition to the grading system or prognostic significance, the cut-off value of MIB-1 LI for the pattern of failure in GB remains to be examined. Therefore, the present findings should be confirmed by larger-scale and multi-institutional studies with standardized evaluation methods for MIB-1 LI.

The MIB-1 LI had the impact in prediction of POF when sites of recurrence were stratified to the central versus others as shown in the Table 3. When the stratification was converted to the subgroups of the central or the in-field versus of the marginal or the distant, the value was not significant (P = 0.23). That might suggest that the MIB-1 with a cut-off = 30 was closely involved the in-field recurrence. Of course, other factors, such as definition of GTV (pre-surgical or post-surgical), margins for CTV, and total dose, should be standardized before proposing the association between the MIB-1 LI and the in-field recurrence, this result need to be clarified in the future study.

It is difficult to state distinct association between radiosensitivity and the MIB-1 LI in GB. As far as we investigated, there are no reports elucidating the direct link in GB. Possible explanation might be that, not only the MIB-1 LI, GB has been reported to contain several abnormalities of molecular parameters, including 1p19q deletion status [21,22], isocitrate dehydrogenase (IDH) genes mutation status [23,24], or methylation status of the O6-methylguanine-DNA methyltransferase gene MGMT promoter methylation status [25-27]. On the contrary, increased cell cycling and MIB-1 LI suggest more aggressive behavior of promote tumor progression and metastasis in variety of cancers [28,29]. Therefore, the MIB-1 with association with other molecular parameters might reflect tumor behavior and POF in patients with GB. Although the MIB-1 LI could be a useful tool to recognize disease characteristics including POF, further clinical and experimental studies are warranted.

From our result, it is possible to propose that patients with low MIB-1 LI have a benefit to receive increased dose of radiotherapy because more often central recurrence might occur. On the contrary, patients with the high MIB-1 LI might not have a benefit because prediction of sites of recurrence were difficult. Therefore, in those patients with the high MIB-1 LI, more aggressive chemotherapy seemed to be more important instead of a dose escalation of radiotherapy. Thus, the MIB-1 LI could be a useful tool to determine individual strategy in patients with GB with prediction of POF.

Among other biomarkers for GB, MGMT methylation seems to be the most intensively examined biomarker [14,16]. Brandes et al. [16] demonstrated that recurrence is correlated with MGMT methylation status in patients who received chemoradiation with temozolomide. Interestingly, Minniti et al. [14] also reported that patterns of recurrence were significantly different according to the methylation status of the MGMT promoter. Recurrences were central/in-field and distant in 64% and 31% of methylated patients versus 91% and 5.4% of unmethylated patients (P = 0.01), respectively. The present study did not include an evaluation of MGMT; however, the correlation between MIB-1 LI and MGMT or other markers and their combination should be examined in future studies. The combination efficacy between MIB-1 LI and MGMT could also affect the strategy for GB treatment.

The MIB-1 LI has been used as a marker of the proliferation rate of various intracranial and extracranial tumors. Okita and coworkers [30] reported that MIB-1 LI of the recurrent tumors identified as a significant independent prognostic factor in their multivariate analysis. Kim and coworkers [31] reported that the values of MIB-1 LI between primary and recurrent tumors were different, and that the MIB-1 LIs of the recurrent tumors were reduced in 75% of patients with GB. Their explanation to the result was that radiotherapy and/or chemotherapy might suppress proliferation, actively proliferating tumors, and they suggest that the MIB-1 LI may play a diagnostic role in recurrent GB. Thus, the prediction of POF by the MIB-1 LI will lead to evaluation and comparison between primary and recurrent tumors, and possibly might illustrate tumor characteristics of GB in the future.

## Conclusions

The proportions of recurrence sites in our series of AA and GB were consistent with previous reports addressing patterns of failure in high-grade gliomas. Our novel findings identified MIB-1 LI, but not treatment factors, as a biomarker that was significantly correlated with the pattern of failure in GB patients. Although the present findings need to be confirmed by larger-scale studies, this report provides important information to help elucidate the biological nature of GB.

## Competing interest

The authors declare that they have no competing interests.

## Authors’ contributions

Conception and design: KU, RS, Provision of study materials or patients: TaS, HN, MN, ToS, KS, EK, Collection and assembly of data: KU, TaS, DM, OF, TS, Data analysis and interpretation: KU, TaS, DM, HN, KY, YO, NM, HA, RS. Manuscript writing: KU, RS. Final approval of manuscript: KU, TaS, DM, HN, KY, YO, NM, HA, MN, OF, ToS, KS, EK and RS. All authors read and approved the final manuscript.

## References

[B1] Stupp R Mason WP van den Bent MJ Weller M Fisher B Taphoorn MJ Belanger K Brandes AA Marosi C Bogdahn U Curschmann J Janzer RC Ludwin SK Mirimanoff RO Radiotherapy plus concomitant and adjuvant temozolomide for glioblastoma N Engl J Med 2005 352 987 996 10.1056/NEJMoa043330 15758009

[B2] Halperin EC Bentel G Heinz ER Burger PC Radiation therapy treatment planning in supratentorial glioblastoma multiforme: An analysis based on post mortem topographic anatomy with CT correlations Int J Radiat Oncol Biol Phys 1989 17 1347 1350 10.1016/0360-3016(89)90548-8 2557310

[B3] Chang EL Akyurek S Avalos T Rebueno N Spicer C Garcia J Famiglietti R Allen PK Chao KS Mahajan A Woo SY Maor MH Evaluation of peritumoral edema in the delineation of radiotherapy clinical target volumes for glioblastoma Int J Radiat Oncol Biol Phys 2007 68 144 150 10.1016/j.ijrobp.2006.12.009 17306935

[B4] Liang BC Thornton AF Sandler HM Greenberg HS Malignant astrocytomas: Focal tumor recurrence after focal external beam radiation therapy J Neurosurg 1991 75 559 563 10.3171/jns.1991.75.4.0559 1653309

[B5] Lee SW Fraass BA Marsh LH Herbort K Gebarski SS Martel MK Radany EH Lichter AS Sandler HM Patterns of failure following high-dose 3-D conformal radiotherapy for high-grade astrocytomas: A quantitative dosimetric study Int J Radiat Oncol Biol Phys 1999 43 79 88 10.1016/S0360-3016(98)00266-1 9989517

[B6] Chan JL Lee SW Fraass BA Normolle DP Greenberg HS Junck LR Gebarski SS Sandler HM Survival and failure patterns of high-grade glioma after three-dimensional conformal radiotherapy J Clin Oncol 2002 20 1635 1642 10.1200/JCO.20.6.1635 11896114

[B7] Nakagawa K Aoki Y Fujimaki T Tago M Terahara A Karasawa K Sakata K Sasaki Y Matsutani M Akanuma A High-dose conformal radiotherapy influenced the pattern of failure but did not improve survival in glioblastoma multiforme Int J Radiat Oncol Biol Phys 1998 40 1141 1149 10.1016/S0360-3016(97)00911-5 9539570

[B8] Jeremic B Grujicic D Antunovic V Djuric L Stojanovic M Shibamoto Y Hyperfractionated radiation therapy (HFX RT) followed by multiagent chemotherapy (CHT) in patients with malignant glioma: a phase II study Int J Radiat Oncol Biol Phys 1994 30 1179 1185 10.1016/0360-3016(94)90326-3 7961028

[B9] Mehta MP Masciopinto J Rozental J Levin A Chappell R Bastin K Miles J Turski P Kubsad S Mackie T Stereotactic radiosurgery for glioblastoma multiforme: Report of a prospective study evaluating prognostic factors and analyzing long-term survival advantage Int J Radiat Oncol Biol Phys 1994 30 541 549 10.1016/0360-3016(92)90939-F 7928484

[B10] Johannessen AL Torp SH The clinical value of Ki-67/MIB-1 labeling index in human astrocytomas Pathol Oncol Res 2006 12 143 147 10.1007/BF02893360 16998593

[B11] Ralte AM Sharma MC Karak AK Mehta VS Sarkar C Clinicopathological features, MIB-1 labeling index and apoptotic index in recurrent astrocytic tumors Pathol Oncol Res 2001 7 267 278 10.1007/BF03032383 11882906

[B12] Ho DM Hsu CY Ting LT Chiang H MIB-1 and DNA topoisomerase II alpha could be helpful for predicting long-term survival of patients with glioblastoma Am J Clin Pathol 2003 119 715 22 10.1309/UN4WV65UH94JEWUV 12760291

[B13] McDonald MW Shu HK Curran WJ Crocker IR Pattern of failure after limited margin radiotherapy and temozolomide for glioblastoma Int J Radiat Oncol Biol Phys 2011 79 130 136 10.1016/j.ijrobp.2009.10.048 20399036

[B14] Minniti G Amelio D Amichetti M Salvati M Muni R Bozzao A Lanzetta G Scarpino S Arcella A Enrici RM Patterns of failure and comparison of different target volume delineations in patients with glioblastoma treated with conformal radiotherapy plus concomitant and adjuvant temozolomide Radiother Oncol 2010 97 377 381 10.1016/j.radonc.2010.08.020 20855119

[B15] Milano MT Okunieff P Donatello RS Mohile NA Sul J Walter KA Korones DN Patterns and timing of recurrence after temozolomide-based chemoradiation for glioblastoma Int J Radiat Oncol Biol Phys 2010 78 1147 1155 10.1016/j.ijrobp.2009.09.018 20207495

[B16] Brandes AA Tosoni A Franceschi E Sotti G Frezza G Amistà P Morandi L Spagnolli F Ermani M Recurrence pattern after temozolomide concomitant with and adjuvant to radiotherapy in newly diagnosed patients with glioblastoma: correlation With MGMT promoter methylation status J Clin Oncol 2009 27 1275 1279 10.1200/JCO.2008.19.4969 19188675

[B17] Heesters MA Koudstaal J Go KG Molenaar WM Analysis of proliferation and apoptosis in brain gliomas: Prognostic and clinical value J Neurooncol 1999 44 255 266 10.1023/A:1006398613605 10720205

[B18] Scott JN Rewcastle NB Brasher PM Fulton D MacKinnon JA Hamilton M Cairncross JG Forsyth P Which glioblastoma multiforme patient will become a long-term survivor? a population-based study Ann Neurol 1999 46 183 188 10.1002/1531-8249(199908)46:2<183::AID-ANA7>3.0.CO;2-7 10443883

[B19] Shibamoto Y Yamashita J Takahashi M Yamasaki T Kikuchi H Abe M Supratentorial malignant glioma: an analysis of radiation therapy in 178 cases Radiother Oncol 1990 18 9 17 10.1016/0167-8140(90)90018-R 2163064

[B20] Hsu CY Ho DM Yang CF Chiang H Interobserver reproducibility of MIB-1 labeling index in astrocytic tumors using different counting methods Mod Pathol 2003 16 951 957 10.1097/01.MP.0000084631.64279.BC 13679460

[B21] Durand K Guillaudeau A Pommepuy I Mesturoux L Chaunavel A Gadeaud E Porcheron M Moreau JJ Labrousse F Alpha-internexin expression in gliomas: relationship with histological type and 1p, 19q, 10p and 10q status J Clin Pathol 2011 64 793 801 10.1136/jcp.2010.087668 21653654

[B22] Malkoun N Chargari C Forest F Fotso MJ Cartier L Auberdiac P Thorin J Pacaut C Peoc'h M Nuti C Schmitt T Magné N Prolonged temozolomide for treatment of glioblastoma: preliminary clinical results and prognostic value of p53 overexpression J Neurooncol 2012 106 127 133 10.1007/s11060-011-0643-0 21725801

[B23] van den Bent MJ Dubbink HJ Marie Y Brandes AA Taphoorn MJ Wesseling P Frenay M Tijssen CC Lacombe D Idbaih A van Marion R Kros JM Sanson M IDH1 and IDH2 mutations are prognostic but not predictive for outcome in anaplastic oligodendroglial tumors: a report of the European Organization for Research and Treatment of Cancer Brain Tumor Group Clin Cancer Res 2010 16 1597 1604 10.1158/1078-0432.CCR-09-2902 20160062

[B24] Ducray F Mokhtari K Crinière E Idbaih A Marie Y Dehais C Paris S Carpentier C Dieme MJ Adam C Hoang-Xuan K Duyckaerts C Delattre JY Sanson M Diagnostic and prognostic value of alpha internexin expression in a series of 409 gliomas Eur J Cancer 2011 47 802 808 10.1016/j.ejca.2010.11.031 21194923

[B25] Hegi ME Diserens AC Gorlia T Hamou MF de Tribolet N Weller M Kros JM Hainfellner JA Mason W Mariani L Bromberg JE Hau P Stupp R MGMT gene silencing and benefit from temozolomide in glioblastoma N Engl J Med 2005 352 997 1003 10.1056/NEJMoa043331 15758010

[B26] Rivera AL Pelloski CE Gilbert MR Colman H De La Cruz C Sulman EP Bekele BN Aldape KD MGMT promoter methylation is predictive of response to radiotherapy and prognostic in the absence of adjuvant alkylating chemotherapy for glioblastoma Neuro Oncol Engl 2010 12 116 121 10.1093/neuonc/nop020 PMC2940581 20150378

[B27] Minniti G Salvati M Arcella A Buttarelli F D'Elia A Lanzetta G Esposito V Scarpino S Maurizi Enrici R Giangaspero F Correlation between O6-methylguanine-DNA methyltransferase and survival in elderly patients with glioblastoma treated with radiotherapy plus concomitant and adjuvant temozolomide J Neurooncol 2011 102 311 316 10.1007/s11060-010-0324-4 20686820

[B28] Kennedy AS Raleigh JA Perez GM Calkins DP Thrall DE Novotny DB Varia MA Proliferation and hypoxia in human squamous cell carcinoma of the cervix: first report of combined immunohistochemical assays Int J Radiat Oncol Biol Phys 1997 37 897 905 10.1016/S0360-3016(96)00539-1 9128967

[B29] Couture C Raybaud-Diogène H Têtu B Bairati I Murry D Allard J Fortin A p53 and Ki-67 as markers of radioresistance in head and neck carcinoma Cancer 2002 94 713 722 10.1002/cncr.10232 11857304

[B30] Okita Y Narita Y Miyakita Y Ohno M Fukushima S Kayama T Shibui S Pathological findings and prognostic factors in recurrent glioblastomas Brain Tumor Pathol 2012 [Epub ahead of print] 10.1007/s10014-012-0084-2 22331317

[B31] Kim JH Bae Kim Y Han JH Cho KG Kim SH Sheen SS Lee HW Jeong SY Kim BY Lee KB Pathologic diagnosis of recurrent glioblastoma: morphologic, immunohistochemical, and molecular analysis of 20 paired cases Am J Surg Pathol 2012 36 620 628 10.1097/PAS.0b013e318246040c 22441548

